# Identification and Quantification of Texture Soy Protein in A Mixture with Beef Meat Using ATR-FTIR Spectroscopy in Combination with Chemometric Methods

**DOI:** 10.22037/ijpr.2019.111580.13242

**Published:** 2019

**Authors:** Zahra Keshavarzi, Sahar Barzegari Banadkoki, Mehrdad Faizi, Yalda Zolghadri, Farshad H Shirazi

**Affiliations:** a *Department of Toxicology and Pharmacology, School of Pharmacy, Shahid Beheshti University of Medical Science, Tehran, Iran. *; b *Pharmaceutical Sciences Research Center, Shahid Beheshti University of Medical Sciences, Tehran, Iran. *; c *Division of Pharmacology and Toxicology, Department of Basic Sciences, School of Veterinary Medicine, Shiraz University, Shiraz, Iran.*

**Keywords:** Meat, Textured soy protein, Food Adulteration, Fourier-transform infrared spectroscopy, Multivariate analyses

## Abstract

Meat, as an important source of protein, is one of the main parts of many people’s diet. Due to economic interests and thereupon adulteration, there are special concerns on its accurate labeling. In this study Fourier transform infrared (ATR-FTIR) spectroscopy combined with chemometric techniques (principal component analysis (PCA), artificial neural networks (ANNs), and partial least square regression (PLS-R)) were employed for discrimination of pure beef meat from textured soy protein plus detection and quantification of texture soy protein in a mixture with beef meat. Spectral preprocessing was carried out on each spectra including Savitzki-Golay (SG) smoothing filter, Standard Normal Vitiate (SNV), scatter correction (MSC), and min-max normalization. Spectral range 1700–1071 cm^-1 ^was selected for further analysis. Principal component analysis showed discrete clustering of pure samples. In the next step, supervised artificial neural networks (ANNs) were performed for classification and discrimination. The results showed classification accuracy of 100% using this model. Furthermore, PLS-R model correlated the actual and FTIR estimated values of texture soy protein in beef meat mixture with coefficient of determination (R^2^) of 0.976. In conclusion, it was demonstrated that ATR-FTIR spectroscopy along with PCA and ANNs analysis might potentially replace traditional laborious and time-consuming analytical techniques to detect adulteration in beef meat as a rapid, low cost, and highly accurate method.

## Introduction

Beef meat is an important but relatively expensive source of protein in food diet. In recent years, due to economic interests, the prevalence of meat fraud and mislabeling has increased. Therefore, many consumers are worried about the meat they eat. The identifying and quantification of adulteration are important for fare trade and protect the health and rights of consumers (1-4) (Lamyaa, 2013). Substitution of high value ingredients with cheaper ingredients frequently happens, especially in meat products, since morphological characteristics disappear in meat products. In fact, replacement of beef or lamb meat with texture soy protein is widely common (5).

There are several analytical methods that have been applied to identify meat species. All of them are DNA or protein -based approaches including polymerase chain reaction, chromatography, mass spectrometry, or electronic spin. However, these methods are expensive and require expert technicians and intricate sample preparation. Recently, innovative and non-destructive Fourier-transformed infrared (FTIR) spectroscopy has been widely investigated as an analytical technique in food analysis and food labeling. This technique is sensitive, rapid, and nondestructive and does not need any complicated sample preparation (6-11). 

Nonetheless, in complex reaction samples, like meat mixtures, serious spectral interferences (or overlapped signals) may result in non-linear correlations between the measured signal and the property of interest. Thus, multivariate data analysis is critically essential. FTIR and chemometric techniques are capable of food identification in a qualitative and quantitative manner. In addition, it is practical to specify the authenticity and the degree of adulteration in food industry, based on the spectral characteristics of the matrix (12, 13). Chemometrics overcomes the limitations of univariate statistics using experimental design, multivariate classification, and multivariate calibration that are achieved by mathematical and statistical techniques alongside computer science to select the best experimental design and treatment of chemical analysis data (14-16). There are several chemometric methods applied to spectroscopy, including partial component analysis (PCA), partial least squares regression (PLS-R), discriminant analysis, artificial neural network (ANN), and principal least squares discriminant analysis. The different goals trailed in spectroscopy determine the suitable choice among these methods (10, 15 and 17-19).

Several studies have tried to detect meat adulteration using FTIR coupled with chemometric methods. For instance, Mid-infrared spectroscopy with PLS-R and soft independent modeling of class analogies (SIMCA) was used for the detection of adulterants in minced beef (6). Furthermore, FTIR spectroscopy combined with PLS, is often used for quantitative analysis of foodstuff (8). In two studies FTIR was used to discriminate between pure beef and beef mixed with offal (heart, tripe, kidney, and liver) (20, 21). To the best of our knowledge, no prior study has enabled discrimination of beef meat, and texture soy protein by FTIR. Although Ofelia and colleagues have used SIMCA to distinguish between ATR spectra of minced beef and the adulterants (horse meat, fat beef trimmings, and soy protein) (6).

In this work, FTIR-ATR spectroscopy coupled with PCA and ANNs is used for discrimination and classification of the pure beef from the texture soy protein with complicated spectra preprocessing. In addition, the use of PLS-R to detect and quantify the presence of texture soy protein in beef mixture is investigated.

## Experimental

*Samples preparation*

Round (7 samples), sirloin (6 samples) and chuck (7 samples) of beef meat was purchased from different local butcheries in Tehran in order to consider the variations among meat composition. Textured soy protein (20 samples) was obtained from different local retail supermarkets. All beef meat samples were transferred to and kept in 4 °C refrigerator before investigation. All samples (each 100 g) were minced and homogenized using a food processor for 2 min, which was carefully washed between each preparation, using 0.2% Triton-X 100 solution followed by distilled water. Then, 10 g of homogenized meat was dried in Petri dishes using vacuum pump for 60 min. Fifty gram of textured soy protein was rehydrated by allowing it to completely absorb twice its weight distilled water in room temperature. The preparation was similar to the meat samples afterwards and homogenization and drying were applied on it. For classification step, 70% of each pure sample was used for training and the rest of the samples were exploited for validation (test data).

*Preparation of adulterated samples for PLS-R model*

A set of reference data (training data) consisting of the texture soy protein and beef meat (equal proportions of Round, sirloin, and chuck) were prepared by mixing texture soy protein with beef meat at concentration ranges of 0, 10, 20, 40, 60, 80, 90, and 100% (w/w). 

The calibration set which included 42 samples (reference data) was produced as follows: 7 repeats for each of six mixtures with stated percentages (6 × 7 = 42). Additionally, a set of 6 independent mixtures in three repeats (6 × 3 = 18) was prepared for using as external validation (test data). 

*ATR-FTIR spectra acquisition*

IR spectra were collected at room temperature using FTIR spectrometer (WQF- 510 Fourier transform spectrometer, Rayleigh Optics, China) equipped with a DLaTGS (deuterated, L-alanine doped triglycine sulfate) detector and ATR crystal (Diamond MIRacle;PIKE Tech- nologies, USA). Measurements were presented in the absorbance mode for the spectral range of 4000 to 400 cm^-1^ with a resolution of 4 cm^-1^. One-hundred scans were co-added to enhance signal to noise ratio (SNR) for each spectrum. The spectra were then subtracted against background air spectrum. The ATR crystal was cleaned after each acquisition using cotton and methanol of analytical grade and dried with lens cleaning tissue.

*Preprocessing step and spectra range selection*

In order to eliminate the effects of noise and nonlinearities of the spectra, preprocessing was applied on raw spectra. Raw data was transferred to mat files using essential FTIR software version 3.00.019, then used as an input spectral data by MATLAB software version 2014b. FTIR spectral data in range of 1700–1071 cm^−1 ^was used for data analysis. Spectra collected in this range were smoothed using Savitzky-golay filter. The data were then preprocessed by scatter correction (MSC), standard normal variant (SNV) and Min-Max normalization.

*Principal component analysis (PCA)*

PCA is a linear unsupervised modern data analysis which is successfully used in meat species determination (22). Reduction of the number of variables (scores), classification, and the representation of a multivariate data table in a low dimensional space are the benefits of PCA application. PCA build a few new variables called principal components (PCs) from linear combination of the main variables therefore, faster and more efficient analysis and classification can be performed (23). The projections of the eigenvector on the PCs are called the scores and are a measure for the similarities among objects that were used for primary inspection of the data set and subsequently for classification of the samples (Riedwyl, 1988). In this work PCA algorithm was used to visualize discrimination between pure beef meat, and textured soy protein samples.

*Artificial neural networks (ANNs)*

To develop and validate the ANNs for discrimination between beef meat and textured soy protein, the MATLAB software version 2014b was used. In this study, a feed forward back-propagation artificial neural network with multilayer perceptron architecture unit was fitted to the training data. Prior to the training process, spectral pre-processing was performed as previously mentioned above. Kurtosis and Sum of High frequency Wavelet (SHW) were selected as a feature of each spectrum and have been used for the input unit. Cross-validation by the random subsets was applied to estimate the performance of the model when applied to new data. Four-layer neural networks were set, containing one output layer, two hidden layers, and one input layer.

*Partial least square regression (PLS-R)*

The partial least squares regression (PLS-R) has the ability to analyze data with strong collinear, noisy, and numerous variables in the predictor matrix X (*i.e.*, independent variables) and responses Y (*i.e.*, dependent variables) (24).

 In this study for quantitative analysis, the obtained spectra of adulterated samples were regressed using PLS-R method. This multivariate calibration analysis, transform the preprocessed FTIR spectra into the latent variables, which are linear combination of original variables with an orthogonal structure, while capturing most of the variance in the original data (25). The training dataset is used to build and train the model and then test data set for determining performance of the model. PLS-R model was created using optimal numbers of the latent variables (5). For the purpose of this evaluation PLS-R method statistical indices were used. Internal cross validation was performed on the same set of spectral data obtained from the standard mixtures that were used to establish the calibration curve. The root mean squared error of cross validation (RMSEcv) was calculated using random subsets of cross validation technique (3 fold). Accuracy of PLS-R model was evaluated by the coefficient of determination (R^2^) of calibration. 

PCA and PLS-R calculations were performed using MATLAB software version 2014b.

## Result and Discussion

*Interpretation of IR spectra*

**Figure 1:**
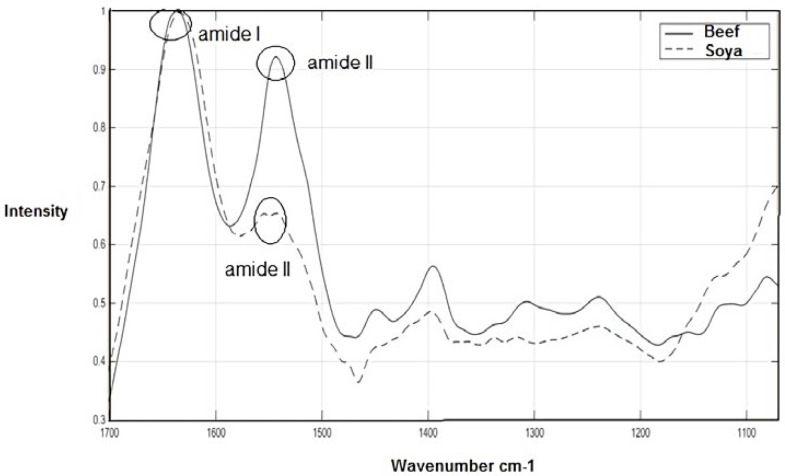
FTIR spectra in the fingerprint region (1700–1071 cm^−1^) for beef and texture soy protein (Soya)

The average FTIR spectra of the beef meat and textured soy protein for the range 1700-1071 cm^-1^ are shown in Figure 1 (pure samples). The fingerprint region 1800 to 950 cm^-1 ^appears to be the major important spectral region which is likely to cover the most variations between biological samples (26, 27). At temperatures above absolute zero, all of the atoms in molecules have molecular vibrations like stretching and bending vibration. When the frequency of a specific vibration is equal to that of the IR radiation, the molecule absorbs the radiation and produces the absorbance bands. Thus, the chemical characteristics of cellular components including protein conformation, glycogen, DNA, lipids, RNA, and content, could be measured by this method (28, 29). 

As expected, in Figure 1, Mean spectra of beef meat and textured soy protein show the amide I bands of the proteins around 1637 cm^−1^ (C–O stretching). Mean Spectra of beef meat shows the band of amide II around 1549 cm^−1^ (N–H bending mixed with C–N stretching). However, as demonstrated in this Figure, the textured soy protein produces small amid II band which poses two peaks. One of the peaks has shifted to high wavenumber and another is shifted to low frequency. 

 The amide I band contains portion from the C=O stretching vibration of the amide group (about 80%) with a lesser portion from the C–N stretching vibration, while the amide II band arise from N–H bending (60%) and C-N stretching (40%) vibration (30). These amide groups are involved in a secondary structure of the proteins especially amide I, because the secondary structural is dependent on characteristic hydrogen bonding pattern between C=O and N-H groups (31). The ratio of amid I to amid II is significantly increased in textured soy protein compared to beef meat which indicated differences in the protein conformation pattern among these samples.

FTIR allows producing great amounts of information for a large number of samples in a relatively short time. This leads to the availability of multivariate data matrices that require the use of mathematical algorithms, for the sake of beneficial use of data. Various models of pattern recognition methods have been used in food science (32). Here we used unsupervised (PCA) and supervised (ANN) pattern recognition techniques for classification.

*PCA results*

In the first step, all data sets (beef and textured soy protein spectra) were preprocessed as mentioned above. Then, principal component analysis was carried out on these data. It has been shown that the discriminatory power of FTIR spectroscopy can significantly be improved using appropriate chemometric methods (33). 

**Figure 2:**
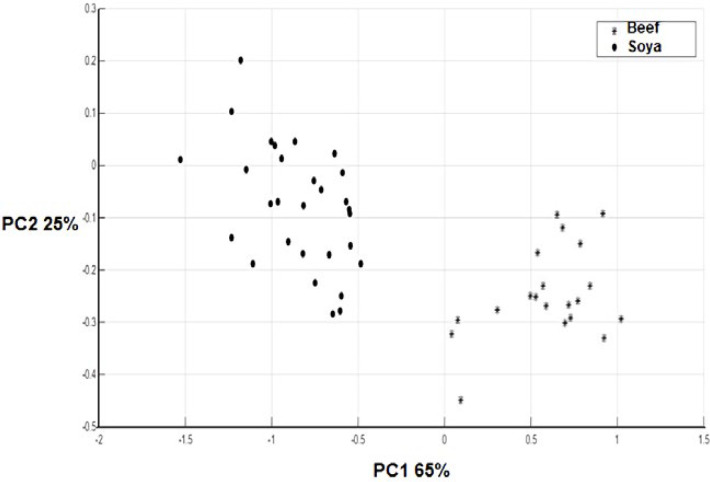
PC scores plot (PCs 1 and 2) of beef meat and texture soy protein (Soya).

As demonstrated in Figure 2 when the scores of beef meat and textured soy protein samples were examined in a two-dimensional plot of the first two principal components (90% of the total variability) a decent separation of the samples into two groups could be attained. It is evident from Figure 1 that the textured soy protein and beef meat have a different spectrum when is compared to each other. In agreement with our study, Ofelia *et al.* has also discriminated the spectra of the minced beef, horse meat, fat beef trimmings, and soy protein at the optimized wave number range of 1800–900 cm^-1^ in a 3D-PCA score plot generated by SIMCA (6). 

*Artificial neural networks (ANNs) results*

ANNs was also applied on the same preprocessing data which were used in PCA. ANNs models originate from an artificial intelligence system, through a supervised machine learning procedure. Each artificial neuron imitates the functioning of a human neuron. Compared to unsupervised learning methods (*e.g.* principal component analysis), supervised learning methods (*e.g.* ANN) have more discriminatory power (33, 34). Back-propagation algorithm is used as a supervised learning algorithm of ANN in the four layered feed-forward networks and this model was trained with Kurtosis and Sum of High frequency Wavelet (SHW) of each spectra as an input data (input neurons).

Our output values in this model were selected to be the mean absolute percentage error (MAPE). During the iterative training process, specific numbers of hidden neurons were selected to minimize global error. Several single ANNs and consecutive combinations of ANNs (modular ANNs) were tested to achieve an optimal network performance. When the model was carried out for the training dataset, beef meat and textured soy protein in the testing dataset were predicted in turn using the learned rules derived from the dataset in model training procedure. In the current study, the classification was carried out using spectral regions at 1700-1071 cm^-1^. The selection of this frequency region was based on its capability to provide the least or no misclassification between two classes. MAPE is zero in all of the runs, which indicates that ANNs are able to classify these two categories with 100% classification accuracy based on the FTIR data set. The results clearly show that the neural networks were able to provide an accurate model to discriminate beef meat from textured soy protein.

*Partial least square regression (PLS-R) results*

The quantitative analysis of textured soy protein samples in beef was performed using partial least square (PLS) regression. The wavenumber region used for quantitative analysis was 1700–1071 cm^−1^. This wavenumber gave the highest coefficient of determination (R^2^) and the lowest values of errors in calibration (RMSEcv) and prediction (RMSEp). This model has R^2^ and RMSEcv values of 0.9761 and 0.78% (w/w) for calibration, respectively. Furthermore, this calibration model was used for the prediction of independent samples which were collected on a different date. The R^2^ and root mean square error of prediction (RMSEP) were 0.9644 and 1.23% (w/w), respectively (Table 1). This result demonstrates that FTIR spectroscopy at wavenumbers of 1700–1071 cm^−1 ^in conjugation with PLS-R model provides accurate results (high R^2^) with ignorable errors (low RMSEcv and RMSEp) for the determination of textured soy protein presence in beef meat mixture. 

**Table 1:** PLS-R performance for analysis of texture soy protein in beef mixture (calibration and validation).

**R2 calibration**	**R2 validation**	**RMSEcv**	**RMSEp**
0.9761	0.9644	0.78%	1.23%

## Conclusion

During the last 40 years, FTIR spectroscopy has been examined for the biological applications using novel hardware and software accessories (35). We used ATR-FTIR spectroscopy as a low-cost and rapid analysis method in combination with multivariate data analysis to detect and quantify the textured soy protein in minced beef. Our results have clearly demonstrated that ATR-FTIR spectroscopy along with PCA and ANNs can be employed to rapidly discriminate pure beef meat from a mixture with textured soy protein. A PLS-R was used to correlate changes with the percentage compositions of texture soy protein in the beef meat. Developed PLS-R models demonstrated high correlations between FTIR data and percentage of texture soy protein in beef meat mixture with low RMSEp for testing the samples.
